# The PEARL toolkit: Using sand flies to identify leishmaniasis animal reservoirs

**DOI:** 10.64898/2026.06.27.734966

**Published:** 2026-06-29

**Authors:** Eva Iniguez, Patrick Huffcutt, Tiago Donatelli Serafim, Pedro Cecilio, Serena Doh, Aaron Pugh, Johannes Doehl, Claudio Meneses, Ben Lambert, Jesus G. Valenzuela, Shaden Kamhawi

**Affiliations:** 1 Vector Molecular Biology Section, Laboratory of Malaria and Vector Research, National Institute of Allergy and Infectious Diseases, National Institutes of Health; Rockville, MD, USA; 2 Vector Biology Section, Laboratory of Malaria and Vector Research, National Institute of Allergy and Infectious Diseases, National Institutes of Health, Rockville, MD, USA; 3 Department of Statistics, University of Oxford, Oxford OX1 3LB, United Kingdom; 4 Pandemic Sciences Institute, University of Oxford, Oxford OX1 3LB, United Kingdom

**Keywords:** leishmaniasis, transmission, reservoirs, blood fed sand flies, molecular tools, gene expression

## Abstract

In many leishmaniasis foci, reservoirs that maintain infection remain unknown. Here, we developed a field-applicable toolkit based on the analysis of individual blood fed sand flies (IBF) to identify reservoirs. Sand flies were given a *Leishmania donovani*-infected first blood meal (iBM1) by feeding artificially on a membrane or naturally on a clinically ill animal followed by two subsequent uninfected blood meals (BMS^+^). Bulk-RNAseq was used to identify two target parasite genes, *sherp* and a novel hypothetical gene (*HPB*), which exhibited a significantly higher expression in BMS^+^ compared to iBM1 sand flies. DNA and RNA were co-extracted from IBF. DNA was used to detect *Leishmania* infection and the blood meal source; RNA was used to assess expression of target genes by qRT-PCR. Linear discriminant analysis (LDA) of target gene expression classified sand fly specimens based on their iBM1 or BMS^+^ status. Co-extraction yielded a mean of >800ng per IBF for DNA and RNA. We detected ^3^1 parasite/s by kDNA qPCR and *ssu rRNA* RT-qPCR. LDA identified iBM1 parasites with a predictive accuracy of ~87% and ~82%, in membrane or naturally fed sand flies, respectively. This toolkit provides an innovative approach to identification of leishmaniasis reservoirs informing targeted control strategies.

## Introduction

Leishmaniasis is a vector-borne disease caused by protozoan *Leishmania* parasites and transmitted by bites of female phlebotomine sand flies during blood feeding. The most prevalent clinical forms are cutaneous (CL) and visceral (VL) leishmaniasis. CL is generally self-healing and affects approximately a million people annually, while VL is responsible for up to 90,000 cases per year and is 95% fatal without treatment^[1]^. Leishmaniasis is mostly a zoonosis, involving animals that remain mostly unknown or unconfirmed, and whose identity is dependent on the nature and biodiversity of disease foci. Of the *Leishmania* species infecting humans, only *Leishmania donovani* is thought to cause anthroponotic visceral leishmaniasis (AVL)^[2]^ though recent studies suggest the likely presence of animal reservoirs^[3, 4]^. These unidentified infection reservoirs persist as one of the most significant knowledge gaps in leishmaniasis foci worldwide, hindering development of efficacious and targeted control strategies to interrupt disease transmission.

Xenodiagnosis is currently the most direct approach to determine the infectiousness of a *Leishmania*-infected host to sand flies and can be used to investigate the role of suspected reservoirs^[3, 5]^. However, this approach is laborious, costly, and selective, making it ineffective and infeasible for establishing the identity of unknown mammalian reservoir hosts in a field-setting. Other molecular biology-based methods are used to screen sand flies for infection or for identification of blood meal sources. However, they lack the sensitivity to distinguish initial parasite stages picked up from a reservoir animal from those residing in the gut during a subsequent blood meal by the vector^[6, 7]^. Sand flies are promiscuous feeders that take blood meals every 5–6 days to support egg development^[8]^. After a sand fly feeds on a disease reservoir, *Leishmania* begins a long developmental cycle that spans multiple blood meals starting with parasite pick-up of amastigotes, followed by differentiation through multiple intermediate stages prior to maturation into, and transmission of infectious metacyclic promastigotes^[8, 9]^. The recurrent natural feeding behavior of sand flies is also critical for parasite development within the midgut and is intimately connected to vector competence and transmission^[9]^. As a result, infected blood fed females collected in disease foci will contain distinct developmental stages of *Leishmania* parasites depending on the maturity of the infection, and the presence of blood alone cannot be used to extrapolate a parasite pick-up event. However, if we can distinguish *Leishmania* forms that are present in the first infected blood meal (iBM1) from those residing in a blood fed midgut during subsequent blood meals (BMS^+^), we can use sand flies to identify disease reservoirs in emerging or established leishmaniasis foci, and is the basis of our approach.

In this study, we developed an innovative field-applicable “Phlebotomines Establish Animal Reservoirs of *Leishmania*” or PEARL toolkit that focuses on in-depth sequential molecular analyses of DNA and RNA co-extracted from individual blood fed sand flies (IBF) to identify leishmaniasis reservoirs. To achieve this, we performed experimental sand fly infections incorporating second and third subsequent blood meals that mimic the parasite life cycle in a natural scenario^[8]^. We used bulk RNAseq from *Leishmania* residing in blood fed midguts to mine targets that can distinguish parasites prevalent in subsequent sand fly feeds from early parasite stages that are present in the initial infected blood meal. Then we then the DNA to detect *Leishmania* parasites and identify the blood meal source in IBF sand flies^[6, 10]^, and RNA to distinguish parasites in iBM1 from those in BMS^+^. Naturally, and without manipulation, this toolkit is tailored to point to leishmaniasis reservoirs by looking beyond an infection with *Leishmania* to specific parasite stages developing in the midgut in the context of the sand fly recurrent blood feeding behavior.

## Methods

### Animals

Male Golden Syrian hamsters (4 to 6 weeks old) were purchased from Charles River Laboratories. Female BALB/c mice (3 to 6 weeks old) were purchased from Jackson Laboratories. All animals were kept at the National Institute of Allergy and Infectious Diseases (NIAID) Twinbrook animal facility in Rockville, Maryland. The NIAID Animal Care and Use Committee reviewed and approved all animal experiments under animal protocol LMVR4E. The NIAID DIR Animal Care and Use Program is fully compliant with the Guide for the Care and Use of Laboratory Animals and with the NIH Office of Animal Care and Use and Animal Research Advisory Committee guidelines. Detailed NIH Animal Research Guidelines can be accessed at https://oma1.od.nih.gov/manualchapters/intramural/3040-2/.

### Parasites

*Leishmania donovani* (MHOM/SD/62/1S strain) parasites were maintained by serial passaging through Golden Syrian hamsters. Amastigotes were harvested from infected hamsters^[11]^ and cryopreserved until use. Tissue-amastigotes were either used to spike defibrinated animal blood for sand fly infections or cultured to generate standard curves. Parasites were cultured at 26°C in Schneider’s insect medium (Gibco) supplemented with 20% heat-inactivated fetal bovine serum, 2 mM L-glutamine (Gibco), 100 U/mL penicillin and 100 μL/mL streptomycin (Gibco).

### Animal infections

Male Golden Syrian hamsters were infected intravenously with 10^7^ purified *L. donovani* metacyclic promastigotes^[11]^. Hamsters were monitored for clinical symptoms of VL according to the LMVR4E animal protocol. Six to ten months later, symptomatic animals were exposed to sand flies or used to harvest tissue amastigotes from the spleen.

### Sand fly infections and subsequent blood meals

*Lutzomyia longipalpis* female sand flies (4–6 days old; Jacobina strain) were used for all infections. Rearing was performed at the Laboratory of Malaria and Vector Research, NIAID, NIH insectary. Sand flies were infected either artificially using a chicken-skin membrane feeder containing animal blood spiked with tissue-harvested amastigotes (5×10^6^ per mL)^[9]^, or naturally by feeding on a *L. donovani*-infected animal^[11]^. Blood fed sand flies were sorted out and maintained at 26°C with 30% sucrose. After the sand flies had taken their first infected blood meal (iBM1), either artificially or via an infected animal, subsequent blood meals (BM2, day 5–6; BM3, day 10–12) were provided by feeding the sand flies for 1–2 hours on an anesthetized (ketamine/xylazine, 100 mg/kg and 10 mg/kg) uninfected mouse. The midguts of infected blood fed sand flies were dissected at discrete timepoints to assess the infection status and maturity as described^[9]^. As blood feeding takes 1–2 hours, our earliest timepoint is indicated as ≤2h throughout the manuscript.

### Preservation of individual blood fed sand flies on Whatman 903 Protein Saver Cards

After each blood-meal event (iBM1, BM2, and BM3) blood fed sand flies were dissected ≤2 to 48 hours and preserved individually using a field-friendly approach. The midgut was dissected by detaching the head and cutting the last three segments of the abdomen below the blood bolus, then gently pulling the entire gut out of the abdomen. The intact midgut was then transferred to a Whatman 903 Protein Saver card (Cytiva) for DNA and RNA preservation^[12]^. Using a DNA/RNase free pipette tip, the midgut was spread on the card and allowed to air dry overnight. The next day, the sample was stored in an individually sealed plastic bags at room temperature, for up to 9 months. All dissecting pins used were first bleached (10%) and then rinsed twice with water before each dissection to avoid carry-over contamination between samples.

### DNA and RNA co-extraction from individual blood fed sand flies

DNA and RNA were co-extracted from preserved blood fed sand flies. Briefly, the dried blood fed midgut was punched using a 3 mm disposable biopsy punch and placed in a 1.5 mL Eppendorf tube. Nucleic acids were co-extracted using the Quick-DNA/RNA Miniprep kit (Zymo). The punched midgut was digested by adding 30 μL of DNA/RNA shield, 15 μL of proteinase K, and 30 μL of PK Digestion Buffer, followed by incubation at 56°C for 2h, with vortexing performed every hour. Nucleic acids were extracted following the manufacture’s tissue protocol, including DNase I treatment of RNA to remove residual DNA. Samples were eluted to a final volume of 100 μl, with the eluate passed through the column twice to improve the yield. Alternatively, this protocol was used successfully for blood fed midguts preserved in 30 μL of DNA/RNA shield (Zymo). Sample concentrations were measured using a Nanodrop and samples were stored at −80°C until further processing.

### Leishmania probe-based kDNA qPCR

We followed our contamination mitigation protocol as previously described to avoid carry-over contamination^[6]^. *Leishmania* kinetoplast minicircle DNA was amplified by a highly sensitive probe-based qPCR using the primers JW11 (5’-CCTATT TTACACCAA CCCCCAGT-3’) and JW12 (5’-GGGTAGGG GCGTTCTGCGAAA-3’) and the TaqMan probe (5’-[Aminoc6+TxRed] RAAARKKVRTRCAGAAAYCCCGT [BHQ2]-3’). Forty nanograms of DNA template, 0.12 μM of forward and reverse primers, and 0.12 μM of the probe were mixed with 12.5 μL Premix Ex Taq probe master mix (Takara) to a final volume of 25 μL and amplified as described^[13]^. DNA from a negative uninfected blood fed midgut and a standard curve were run on each plate. Standard curves of DNA were generated by spiking an uninfected blood fed sand fly midgut with 10^5^ *L. donovani* parasites, followed by DNA extraction. A 10-fold serial dilution was then performed on DNA from an uninfected blood fed midgut. The standard curve was used for parasite quantification by fitting the cycle threshold (CT) of experimental samples into the equation of the line. Each sample was run in duplicate or triplicates using a CFX96 Touch Real-Time PCR System (Bio-Rad).

### Bulk RNA sequencing in infected blood fed sand flies

For the identification of target genes expressed by the different *Leishmania* stages encountered in the blood fed midgut after a first infected blood meal (iBM1) and in subsequent blood meals (BMS^+^), *Lu. longipalpis* sand flies were infected with *L. donovani* using a membrane feeder. Pools of 20 midguts were dissected at 48 hours after iBM1, and pools of 5 midguts were dissected at 0, 8, 24, and 48 hours after BM3. To capture the retroleptomonad stage^[9]^, a BM3 was given at day 10, after confirmation of the presence of infectious metacyclic promastigotes. Pools of midguts were preserved in 30 μL DNA/RNA shield (Zymo). RNA was extracted following the tissue protocol of the RNeasy Mini Kit (Qiagen) and submitted for library preparation and sequencing using the NovaSeq6000 Illumina platform (Novogene).

To recover parasite-derived reads specifically while preventing vector transcripts from being mis-assigned to parasite genes, bulk RNA-seq reads were aligned by HISAT2 against a single concatenated reference combining the *Lu. longipalpis* and *L. donovani* genomes. Only *L. donovani* gene-level raw counts were retained for downstream differential-expression analysis. Because the libraries were generated from this mixed vector–parasite genetic material, and because iBM1 specimens contain far fewer parasites than amplified infections following subsequent blood meals (BMS^+^)^[9]^, samples were first evaluated for parasite transcriptome adequacy. For each sample, parasite-aligned fragments were calculated as the sum of counts across parasite genes, and the transcriptome breadth as the number of parasite genes detected at counts per million (CPM) ≥ 1. Samples were retained only if they met both criteria: ≥ 10,000 parasite fragments and ≥ 300 parasite genes detected at CPM ≥ 1. In practice, all retained/excluded decisions were driven by the fragment-count threshold, as every sample satisfied the gene-detection criterion. Parasite counts below the fragment threshold were dominated by sampling zeros that destabilize dispersion estimation. This criterion retained the iBM1 48-hour timepoint and all BM3 timepoints, each with three biological replicates. Retained iBM1 samples recovered ~6,850–7,450 detected parasite genes, approaching the full *L. donovani* coding complement and providing sufficient transcriptome breadth for differential expression despite lower sequencing depth. We retained low-depth iBM1 samples deliberately rather than excluding them under conventional depth-based quality control. Low parasite burden is the defining biological feature of the early stage of an infection (iBM1), and the precise field condition the toolkit is aiming to detect; discarding these samples would discard the target signal. To guard against sparsity-driven false positives, all candidate transcripts were subsequently validated by RT-qPCR in independent membrane fed and naturally fed sand flies.

Differential expression analysis was performed in R using DESeq2^[14]^. Genes with fewer than 10 summed counts across retained samples were removed. Size factors were estimated using the post-counts method to accommodate the zero-inflation inherent to parasite-only count matrices from vector tissue. The primary comparison contrasted iBM1pp against BM3pp; within-BM3 temporal dynamics were additionally modeled across the retained timepoints. Log_2_ fold-change estimates were shrunken using apeglm^[15]^ to stabilize effect sizes for lowly-expressed genes while preserving large differences. Top candidates were selected by filtering first by high statistical significance using the adjusted p value (*p* < 0.05) calculated by Benjamini-Hochberg. Then, the ten most significant and upregulated genes were ranked by the highest log_2_ fold change. The pattern of top ten candidates genes was manually inspected using the counts per million (CPM) plots, which normalize read counts per million mapped reads to allow comparisons across samples from independent experiments.

### Identification of an unannotated *sherp* gene

The *sherp* gene is not annotated in the *Leishmania donovani* BPK282A1 reference genome (TriTrypDB release 68). To recover the gene directly from the genome, the *L. major sherp* protein (LmjF.23.1080) and the *L. infantum sherp* protein (LINF_230018800) were used as queries in a tBLASTn search (NCBI BLAST+ v2.12.0; default parameters with low-complexity filtering disabled) against the *L. donovani* BPK282A1 genome assembly. Both queries identified a single, unambiguous locus on chromosome 23 (Ld23_v01s1, positions 506,195–506,365, minus strand; E-value ≤ 8 × 10^−32^), with no comparable secondary hits elsewhere in the genome (next-best E-value > 2). The corresponding 171-bp coding sequence was extracted and conceptually translated, yielding a 57-residue, strongly hydrophilic, acidic protein consistent with the known *sherp* product. For the read-counting analysis, this locus was added to the *L. donovani* annotation as a gene/mRNA/exon/CDS feature at the identified coordinates prior to read quantification. The gene is otherwise reported by genomic coordinates, as no TriTrypDB or NCBI identifier exists for it in *L. donovani*.

### *sherp* locus architecture and copy number

Because *sherp* lies within the tandemly-arranged *HASP/sherp* gene family, copy number was assessed in both species to ensure unambiguous read assignment. In *L. infantum* JPCM5, two annotated *sherp* copies are present on chromosome 23 (LINF_230018650 at LinJ.23:510,745–510,918 and LINF_230018800 at LinJ.23:514,920–515,093, both minus strand, ~4 kb apart); the two copies are identical at both the protein (57/57 residues) and nucleotide (174/174 bp) level, and each is identical to the *L. donovani* sequence. Copy number in the *L. donovani* BPK282A1 assembly was determined by BLASTn of the *L. donovani sherp* coding sequence against the assembled genome (NCBI BLAST+ v2.12.0; E-value threshold 1 × 10^−5^), which returned a single full-length hit (chromosome 23, 506,195–506,365; 100% identity over 171 bp) with no additional matches. The BPK282A1 assembly therefore represents this locus as a single *sherp* copy, in contrast to the tandem pair resolved in the more contiguous *L. infantum* assembly. As a consequence, RNA-seq reads mapping to *sherp* are assigned unambiguously to the single annotated *L. donovani* feature, and no multi-mapping correction across paralogous copies was required.

### *Quantification of live parasites and expression of* sherp *and* HPB *by One-Step RT-qPCR*

To quantify and confirm the presence of live parasites in a midgut, RNA samples were first screened using primers targeting the *Leishmania* small subunit 18S ribosomal RNA (*ssu rRNA*), previously identified as a housekeeping gene in infected sand flies^[16]^. A standard RNA curve was run in parallel for live-parasite quantification. To construct the standard curve, RNA from a sample with molecular counts (iBM1, 10^3^ parasites) or direct counts (BM2 and BM3, 10^4^ parasites) was used as the highest point on the standard curve (3 replicates). A 10-fold serial dilution was performed into RNA extracted from individual uninfected blood fed midguts. RT-qPCR reactions were prepared by mixing RNA (5 μL) with 10 μL of Luna^®^ Universal One-Step RT-qPCR master mix (NEB) and 0.6 μM of each forward and reverse primers, in a final volume of 20 μL. PCR conditions were 55°C reverse transcription for 10 min, 95°C initial denaturation for 1 min, 40 cycles of 10 s at 95°C, 30 s at 60°C for extension. Primers targeting the 18S ribosomal RNA (*ssu rRNA*), forward (5’-CCATGTCGGATTTGGT-3’) and reverse (5’-CGAAACGGTAGCCTAGAG-3’), were used as previously designed by others for *Leishmania infantum*^[16]^. Primers, forward (5’-GCGGAGACGAGCGACAAT-3’) and reverse (5’- CGAGCCGCCGCTTATCTT-3’), were designed to target the small hydrophilic endo-reticulum associated protein (*sherp*) gene in *L. donovani*, identified previously as a metacyclic promastigote stage-specific gene^[16, 17]^. For this study, forward (5’-GATGAGGACTGCAACACCCA-3’) and reverse (5’-AACGCCACAGGGGTGATAAG-3’) primers were designed to amplify the hypothetical protein (*HPB*) gene mined in our RNAseq data. For each sample, the three targets (*ssu rRNA*, *HPB* and *sherp*) were run in parallel on the same plate. An uninfected blood fed midgut was included as control in each plate. ΔCT for stage enriched genes was calculated by subtracting the mean CT of a negative fed midgut from the mean CT of the infected sample.

### PCR for blood meal identification

To investigate the time post-feeding during which a single or multiple blood meals could be detected in the fed fly, we fed sand flies artificially using a membrane feeder. For a single blood source, we use either human blood (left over samples collected for another study under IRB human protocol 000331-I) or cow blood (Charles River Laboratories). For detection of multiple blood meal sources, sand flies were fed on mixed cow and human blood at 60:40, 75:25, and 90:10 ratios, respectively. Blood fed midguts were collected right after feeding, and every 24 hours for up to 6 consecutive days. DNA was extracted from individual samples as described above and amplified using primers targeting the mitochondrial *cytochrome b* (cow) and *cytochrome c oxidase I* mitochondrial (human) regions, highly conserved among vertebrates^[18]^. The PCR conditions followed were as described^[6]^.

### Classifying iBM1pp from BMS^+^pp

qRT-PCR data from two and three independent experiments were analyzed from artificial- and hamster-initiated infections, respectively. Both datasets were processed with an identical pipeline. Column names were standardized, and the per-target mean cycle threshold (CT) values for the *HPB* and *sherp* assays were cleaned: samples that did not amplify were assigned a CT value of 40, and any CT values exceeding 40 were capped at this level, treating 40 cycles as the assay's limit of detection. Each CT value was then converted to a normalized abundance score as (40 – CT), so that higher values correspond to earlier amplification and greater target signal. The number of blood meals per sample was used to define a binary outcome, classifying each sample as having received a iBM1 or BMS^+^. Data from independent experiments were combined into a single dataset (retaining a timepoint identifier, which was not used as a feature for classification). For each dataset, a linear discriminant analysis (LDA) classifier was trained to distinguish iBM1 from BMS^+^ using the two normalized assay scores (*HPB* and *sherp*) as predictors. Each dataset was partitioned into training (70%) and held-out test (30%) sets using stratified random sampling on the outcome to preserve class balance. Models were specified within the *tidymodels* framework as linear discriminant models using the MASS engine and were fit via a workflow combining this specification with a recipe regressing iBM1 and BMS^+^ on the two predictors. Each fitted model was applied to its corresponding held-out test set, and classification performance was summarized with overall accuracy, F-measure, precision, and recall. The complete workflow was executed in R and was implemented as a reproducible pipeline with the *targets* package.

### Statistical analysis

The Mann-Whitney and Kruskal Wallis tests were performed for comparisons across two or multiple groups, respectively, and are specified in each figure legend. *Spearman* correlation and coefficient of variation (R) was calculated by simple regression to correlate parasite quantification per midgut by direct microscopy counts to molecular counts by kDNA (DNA) and *ssu rRNA* (RNA). Statistical analysis was performed using Graph pad prism (10.4.1). A *p* value of ≤ 0.05 was considered significant.

## Results

To build the toolkit, we first optimized the co-extraction of DNA and RNA from IBF midguts, that were infected by membrane-fed on blood spiked with *Leishmania* parasites (mean 849.2 ng ±SD 246.1 and mean 856.6 ng ±SD 265.2, respectively), or fed on a clinically ill hamster (mean 864.9 ng ±SD 242.5 and mean 1135.0 ng ±SD 317.8), respectively (figure S1A,B), and preserved on Whatman 903 Protein Saver cards. Nucleic acids were successfully extracted from samples collected at ≤2 to 48 hours post-infection with enough material to run several assays even when cards were stored at room temperature for up to 9 months post-collection.

**Figure 1.**
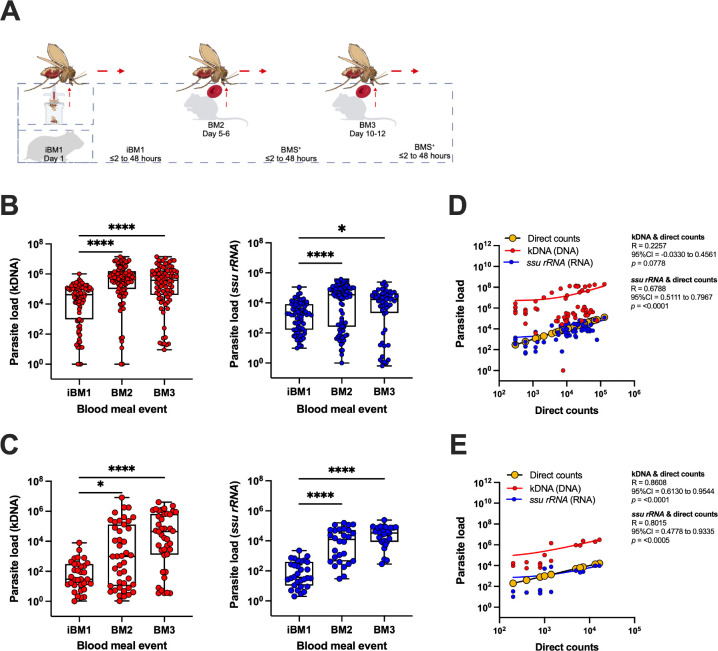
Establishing accurate quantification of *Leishmania* parasites from individual blood fed sand flies. (A) Experimental timeline. Sand flies were infected by membrane feeding on blood spiked with *L. donovani* parasites or on a clinically ill hamster. Sand flies were provided subsequent uninfected blood meals from mice at days 5–6 (BM2) and 10–12 post-infection (BM3). Individual fed midguts were collected from ≤2 to 48 hours after each blood meal event. (B, C) Parasites were molecularly quantified using a probe-based qPCR targeting kinetoplast DNA (kDNA, red circles), or RT-qPCR targeting the constitutively expressed parasite gene, *ssu rRNA* (blue circles), for sand flies fed on a membrane (B) or a clinically ill hamster (C). Cumulative data of two independent experiments are shown. (D, E) Parasite quantification by kDNA qPCR (DNA) and *ssu rRNA* RT-qPCR (RNA) was correlated with direct parasite counts by microscopy for a subset of samples from membrane fed (D) or naturally fed (E) sand flies. A standard curve was included in each plate to calculate parasite load per sample. Bar, mean ±95% CI. Kruskal Wallis test. *Spearman* correlation and coefficient of variation (R) with ±95% CI are provided for two independent experiments (D), or one experiment (E). (B,C) Each data point, mean of an IBF midgut ran in duplicate. iBM1, first infected blood meal; BM2, second uninfected blood meal; BM3, third uninfected blood meal; BMS^+^, subsequent blood meals. A p value of ≤0.05 was considered significant, *p < 0.05, ****p < 0.0001. Created in BioRender. Iniguez, E. (2026) https://BioRender.com/lx0la30. Part of the Illustrations from NIAID Visual & Medical Arts, BioArt Source (bioart.niaid.nih.gov/bioart/000458) (A).

Next, we wanted to establish the accuracy of parasite counts in a blood fed gut after artificial membrane infections or a more natural pick up from a *Leishmania*-infected animal. To mimic their natural feeding behavior, we fed sand flies on blood spiked with *L. donovani* parasites or on a clinically ill hamster (iBM1), followed by two uninfected blood meals (BMS^+^) on mice, given every 5–6 days (BM2 and BM3), and processed the sand flies individually at discrete timepoints ≤2 to 48h after each blood meal (figure 1A). To detect *Leishmania* parasites, we screened the samples with a widely used sensitive Taq-Man probe-based qPCR targeting kinetoplast DNA (kDNA)^[10]^ or by RT-qPCR targeting the constitutive 18S *ssu rRNA* gene^[16]^ (figure S1C,D; figure 1B,C). Amplification of kDNA from membrane fed IBF yielded a median of 42,867 parasites per midgut (IQR 948.3 – 120,724; iBM1), 562,579 (IQR 100,051 – 1,562,882; BM2), and 378,966 (IQR 40,418 – 1,721,649; BM3), representing early, intermediate, and mature infections, respectively (figure 1B). In contrast to kDNA, the expression of the constitutive *ssrRNA* gene in IBF was lower, yielding a median parasite count of 1,938 (IQR 163.7 – 8,392; iBM1), 34,987 (IQR 260.8 −105,597; BM2), and 17,049 (IQR 2,098 – 44,329; BM3) for membrane fed specimens (figure 1B). Interestingly, kDNA amplification from naturally fed IBF had a lower number of parasites per midgut in early (iBM1, median 30.1, IQR 12.3 – 304.7), intermediate (BM2, median 977.4, IQR 10.5 – 130,559.0) and mature (BM3, median 44,108.0, IQR 1,273.0 – 665,933.0) infections when compared to membrane fed sand flies (figure 1B,C), and were comparable to *ssu rRNA* counts for early (iBM1, median 37.5, IQR 10.5 – 400.3), intermediate (BM2, median 12,192, IQR 424.8 – 52,141), and mature (BM3, median 32,602, IQR 8,534 – 76,510) infections (figure 1C). These data demonstrated that parasite count estimates from membrane fed IBF were 3.15-log (kDNA) and 1.71-log (*ssu rRNA*) higher compared to naturally fed (figure 1B,C) sand flies for iBM1, supporting previous findings^[9]^. Additionally, we noted that the increase in IBF parasite counts during subsequent blood meals (BM2 and BM3) were less pronounced for membrane fed compared to naturally fed sand flies, for both kDNA and *ssu rRNA* (figure 1B,C).

Next, to establish the accuracy of parasite quantification using kDNA qPCR or *ssu rRNA* RT-qPCR in IBF, we compared them to direct microscopy counts on a subset of iBM1 and BMS^+^ samples (figure S2; figure 1D,E). Molecular estimates of membrane fed IBF (figure S2A) by kDNA qPCR were weakly correlated (R = 0.2, *p* = 0.08) to direct counts (figure 1D). In contrast, *ssu rRNA* RT-qPCR expression exhibited a strong correlation (R = 0.7, *p* < 0.0001) with parasite counts by microscopy (figure 1D). For IBF fed on a clinically ill animal (figure S2B), direct counts by microscopy significantly correlated not only with *ssu rRNA* (R = 0.8, *p* < 0.0005), but also with kDNA (R = 0.9, *p* < 0.0001; figure 1E), supporting prior conclusions. Collectively, these data indicate that kDNA qPCR may significantly inflate the parasite burden of membrane fed IBF, possibly because of the higher uptake of parasites in iBM1. However, both kDNA qPCR and *ssu rRNA* RT-qPCR provide a more accurate estimate of the parasite burden in naturally fed IBF, with the added benefit of confirming the presence of live parasites by the latter.

We then searched for genes whose expression can distinguish the parasite populations residing in iBM1 (iBM1pp) compared to those prevalent in BM2 and BM3 (BMS^+^pp). Initially, we screened genes previously described in the literature as amastigote-specific, aiming to target the first parasite stage after blood feeding. Our results indicated that the amastin gene family^[19]^, deoxyribose-phosphate aldolase (DERA)^[20]^, LinJ^[21]^ and A2^[22]^ genes are strongly expressed across other parasite stages present in BMS^+^ and are not exclusively expressed in iBM1pp (figure S3A). Additionally, *sherp,* not previously annotated for *L. donovani* (TriTrypDB-68_*L. donovani* BPK282A1) (table 1), and described extensively as a metacyclic promastigote stage-enriched gene in other *Leishmania* species ^[16, 17, 23]^, is weakly expressed in iBM1 early stage parasites, but shows no difference between BM2pp and BM3pp, when there are few-to-no versus numerous metacyclic promastigotes present, respectively (figure S3B).

Because of these results, we decided to search for novel targets using a bulk RNAseq approach. We compared gene expression in RNA extracted from pools of infected blood fed sand flies collected at pertinent timepoints post iBM1 and BM3 given on day 10 post infection (table 2). Supporting our previous findings (figure S3A), amastigote-related genes from the amastin (amastin surface glycoprotein, putative, LdBPK 080650.1) and A2 (5’a2rel-related protein, LdBPK 220660.1; 3'a2rel-related protein, LdBPK 220560.1) families were upregulated in BM3pp and expressed at similar or higher levels than in iBM1pp (figure S4A). As expected, expression of *sherp,* was upregulated in BM3 compared to iBM1 (figure S4B) supporting our earlier findings (figure S3B). Pertinently, we identified a novel late stage-enriched hypothetical protein that we termed hypothetical protein B (*HPB*, LdBPK_262710.1). *HPB* is located on chromosome 26 of *Leishmania donovani* (Ld26_v01s1; start: 1045537, stop: 1046671, length: 1134bp) and is of unknown function. *HPB* gene expression was significantly lower in iBM1 compared to BM3 (adjusted p value = 1.1×10^−13^) (figure S4C). Of note, *HPB* expression was higher than *sherp* across all timepoints (figure S4D). Importantly, sequence alignment of the *HPB* gene shows a high percent of identity to other *Leishmania* species of clinical importance such as *L. infantum* (99.8%), *L. major* (94.6%), and *L. mexicana* (91.8%) (table 3). Similarly, *sherp* and *ssu rRNA* genes also share a high percent identity between *Leishmania* species (table 3). Unfortunately, our search for genes that were significantly upregulated in iBM1 compared to BM3 (such as the top target LdBPK_303240.1), did not perform well when validated by RT-qPCR despite extensive analyses (figure S5A,B).

**Figure 2.**
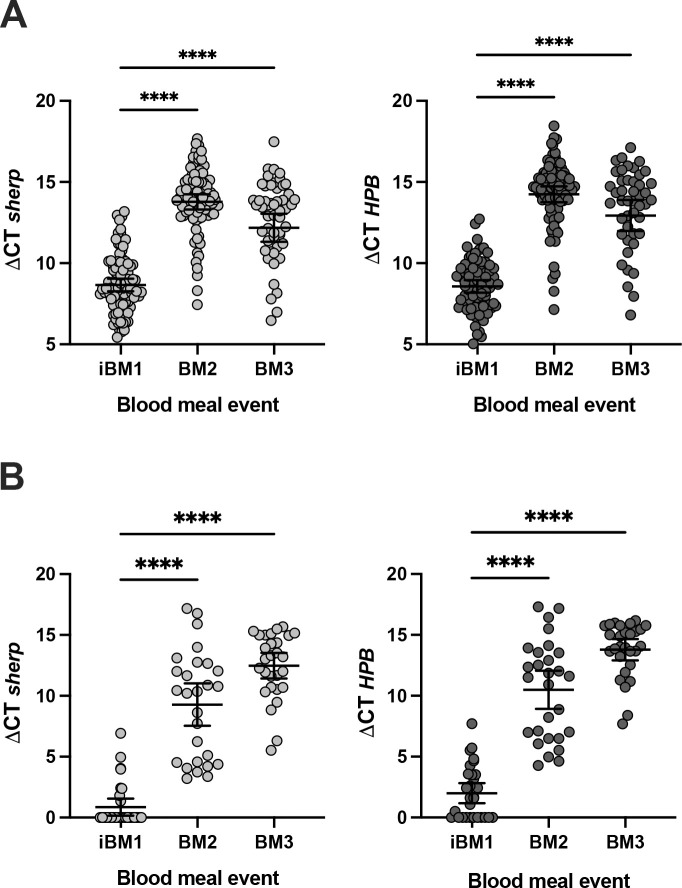
Stage-enriched expression of *Leishmania donovani* target genes distinguishes parasites within the first infected blood meal from those in subsequent blood meals in fed sand flies. (A, B) Expression of stage-enriched *sherp* or *HPB* at each blood meal in membrane fed (A) or naturally fed (B) sand flies. ΔCT for each gene was calculated by subtracting the mean CT value of a midgut fed on uninfected blood from the mean CT value of the infected sample; Bar, mean ± 95% CI are shown; Kruskal Wallis test. Cumulative data of two independent experiments are shown. Each data point represents the average of an IBF midgut ran in duplicate. A p value of ≤0.05 was considered significant, ****p < 0.0001. iBM1, first infected blood meal on day 1; BM2, second uninfected blood meal on day 5–6; BM3, third uninfected blood meal on day 10–12. CT, cycle threshold.

We next validated the expression of *sherp* and *HPB* transcripts in IBF by One-Step RT-qPCR (figure 2). Both stage-enriched genes significantly (*p* < 0.0001) distinguished iBM1pp from BMS^+^pp in membrane-fed sand flies (figure 2A). The ΔCT of *sherp* showed a median expression of 8.4 at iBM1, increasing significantly (*p* < 0.0001) to 14.0 at BM2 and 13.2 at BM3. A similar trend was observed for *HPB*, where the median ΔCT was 8.6 at iBM1, and was significantly higher (*p* < 0.0001) in BM2 (14.7) and BM3 (13.8). Importantly, parasites from naturally fed sand flies also exhibited a lower expression of *sherp* and *HPB* at iBM1 that was significantly (*p* < 0.0001) upregulated in BMS^+^ (figure 2B). For *sherp,* the median ΔCT was 0.0 at iBM1 and increased significantly (*p* < 0.0001) at BM2 (10.4) and BM3 (13.1). Likewise, *HPB* expression had a median ΔCT of 1.6 at iBM1, which increased significantly (*p* < 0.0001) to 11.6 at BM2 and 14.1 at BM3 (figure 2B).

As the PEARL toolkit links iBM1pp to the blood source to indicate the reservoir animal, and sand flies take a blood meal every 5–6 days^[8, 9]^, we wanted to establish the length of time vertebrate host DNA can be detected in blood fed sand flies. We used PCR targeting the vertebrate-specific mitochondrial gene regions, cytochrome b (*cytb*) and cytochrome c oxidase subunit I (COI)^[6]^. Host DNA was detected up to day 4 in sand flies that had fed on cow (*cytb*) or human (COI) blood and had a visible blood bolus when collected, with no detection by day 5 after the blood passed (figure S6A). We also evaluated the sensitivity of this method for detecting DNA from multiple hosts by feeding sand flies on cow and human blood mixed in different ratios (60:40, 72:25, 90:10). DNA from both hosts was detected successfully even for the host with the lowest proportion (figure S6B).

**Figure 3.**
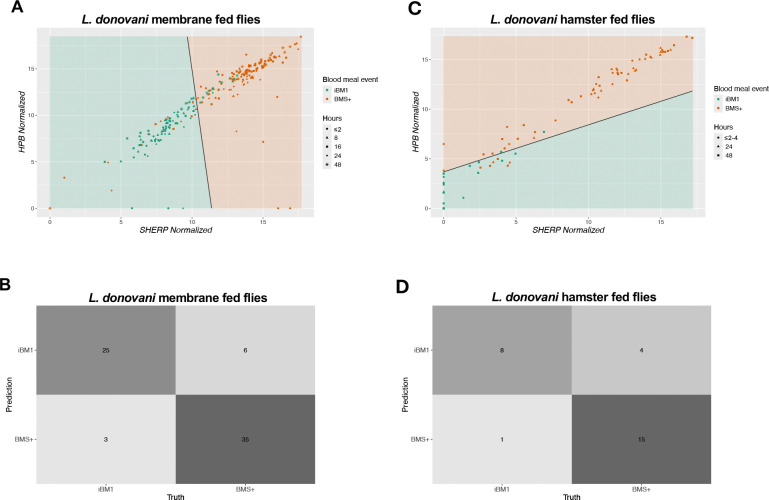
Target gene expression reliably differentiates the first *Leishmania donovani-*infected blood meal from subsequent blood meals in membrane and hamster fed sand flies. (A, B) Linear discriminant analysis using *HPB* and *sherp* as predictors. Black line, boundary determined by LDA fitted to the full dataset. (C, D) Confusion matrices determined by making predictions on an independent test set. iBM1, first infected blood meal. BMS^+^, subsequent blood meals.

We applied a machine learning algorithm to test whether iBM1pp could be differentiated from BMS^+^pp using the *HPB* and *sherp* gene expression obtained by RT-qPCR. By visualizing these bivariate measures and overlaying the number of blood meals taken by the sand fly, BMS^+^ had higher gene expression scores from both of these measures, in both artificially-fed and hamster-fed sand flies (figure 3A,C). This meant that a basic LDA algorithm was able to choose a linear boundary in these spaces that resulted in a relatively clean separation between these two classes. On an independent test set comprising 30% of the data, iBM1pp could be separated from BMS^+^pp with an accuracy of approximately 87% for the artificial feeding experiments, and 82% for the hamster experiments; other metrics summarizing the classification performance are given in table 4, which indicate that both targets when combined outperformed the single target, with similarly reasonable performance.

**Figure 4.**
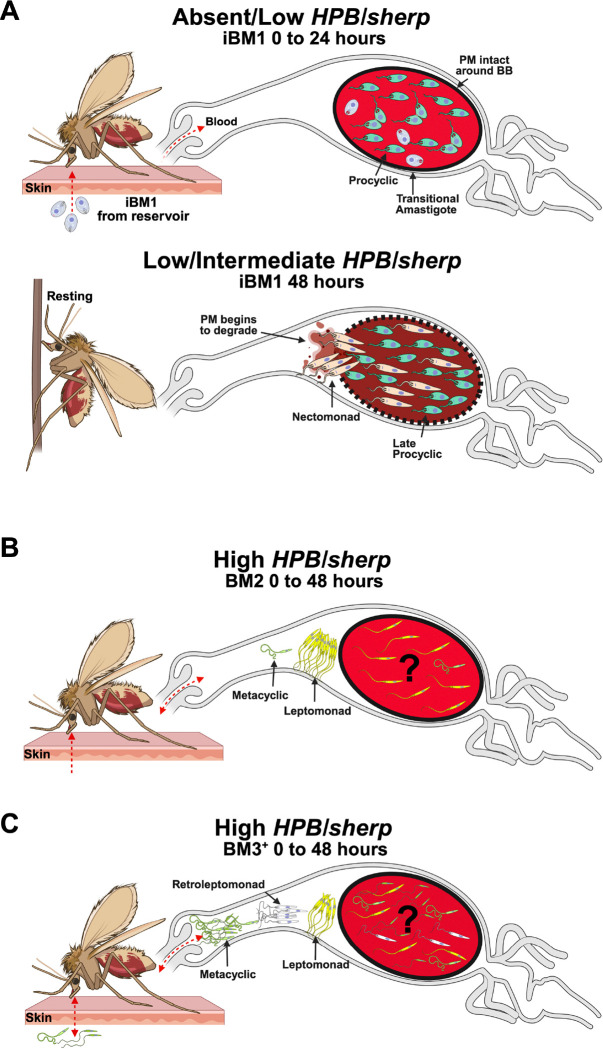
Identifying animal reservoirs by deploying a field-friendly sand fly toolkit in leishmaniasis foci. (A) During the first infected blood meal (iBM1) sand flies ingest amastigotes from an infected reservoir. In the sand fly midgut, amastigotes transition to procyclic promastigotes and divide by 24 hours. At this stage of the infection, the iBM1 parasite population (iBM1pp) is contained within the blood bolus (BB) and protected by the peritrophic matrix (PM). In this scenario *sherp* and *HPB* expression is absent or detected at very low levels (A, top). Around 48 hours after the initial infected blood meal, the PM begins to degrade as blood digestion progresses, permitting nectomonad promastigotes to exit into the lumen and attach to the midgut epithelium (A, bottom). At this timepoint, iBM1pp contains mainly nectomonad forms with few procyclic promastigotes and shows low to intermediate *sherp* and *HPB* expression (A, bottom). (B, C) The *Leishmania*-infected sand fly takes subsequent blood meals (BMS^+^) every 5–6 days. (B) During BM2, leptomonad promastigotes predominate and a few infectious metacyclic promastigotes may appear. (C) During BM3^+^, several parasite forms may be present, including leptomonad, retroleptomonad, and infectious metacyclic. In both BM2pp and BM3^+^pp, *sherp* and *HPB* expression is significantly higher than in iBM1pp (B,C). Upon imbibing a BMS^+^, the location of parasites with respect to the PM remains unknown (?). The most likely scenario is that parasites may reside both inside and outside the BB (B,C). iBM1, BM2, and BM3^+^ depict a blood meal given on day 1 (iBM1), day 5–6 (BM2), or day 10–12 and after (BM3^+^). Created in BioRender. Iniguez, E. (2026) https://BioRender.com/hah62lk. Part of the Illustrations from NIAID Visual & Medical Arts, BioArt Source (bioart.niaid.nih.gov/bioart/290, 291, 294, 296, 458 and 461).

Figure 4 depicts the rationale for our PEARL toolkit which takes into account the multiple feeding behavior of sand flies and the developmental cycle of the *Leishmania* parasite within the blood fed midgut. During its first infected blood meal, the sand fly picks up amastigotes from an infected vertebrate host which start to transition to procyclic promastigotes as early as 5 hours after the blood meal^[24]^ (figure 4A, top). Procyclic promastigotes begin to divide, increasing in number over the first 24 hours of infection^[22]^. In this scenario, *sherp* and *HPB* genes are not detected or detected at very low levels. Based on empirical data, the expression of both target genes by RT-qPCR starts to increase around 48 hours post-iBM1 (figure 4A, bottom). We hypothesize that the emerging nectomonad form begins to express *sherp* and *HPB* as it exits the peritrophic matrix (PM) into the midgut lumen. The infected sand fly then takes BM2 at 5–6 days after its first blood meal (figure 4B), and another (BM3^+^) every 5–6 days (figure 4C). During this timeframe, several parasite stages that may reside within or outside the newly secreted PM, are present including letptomonad, retroleptomonad, and infectious metacyclic promastigotes^[8, 9]^. In this scenario, expression of *sherp* and *HPB* in BMS^+^pp is significantly higher when compared to the iBM1pp (figure 4).

## Discussion

*Leishmania* species that are pathogenic to humans are mostly zoonotic in nature^[25]^, yet the identity of animal reservoirs in many disease foci remains in question^[3, 4, 25]^, posing a serious challenge to control efforts. This is partly because adequate screening of animals, particularly if they are wild and diverse, is difficult and costly. In this study we introduce the PEARL toolkit, a field-friendly progressive set of analyses that determines potential animal reservoirs of leishmaniasis from blood fed female sand flies. As such, PEARL provides an additional and epidemiologically vital piece of information from field-captured *Leishmania*-infected blood fed sand flies^[6, 26]^.

PEARL innovates by its ability to distinguish parasites developing in a first infected blood meal, taken by a sand fly from its reservoir host, from those present in subsequent blood meals, taken by infected females from prevalent animals. We hypothesize that our ability to distinguish iBM1pp from BMS^+^pp is related to the unique environment of the former which resides in a blood bolus taken from the reservoir host. Amastigotes taken up in the blood meal are contained by a PM that separates the parasites from the sand fly midgut. This condition is unique in that the developing parasite population has not yet been in contact with the midgut environment. Our data show that at 48h post-iBM1, which coincides with the initiation of PM degradation^[24, 27]^, the parasites begin to upregulate *HPB* and *sherp* expression, potentially as an adaptation to the new midgut environment. During PM degradation, the parasites escape into the midgut lumen where they interact with epithelium for the duration of the sand fly lifespan. This hypothesis is supported by the unexpected observation that *sherp*, a well-studied gene that has been closely linked to the development of infectious metacyclic promastigotes^[17, 23, 28]^, is highly expressed on day 6 post-infection when this stage is absent. Moreover, *sherp* expression remains constant as the infection matures, and seems to be more related to contact with the midgut environment than to the appearance of infectious metacyclic promastigotes. Additionally, amastigote-associated genes such as the *Amastin* and *A2* genes were strongly expressed by promastigotes developing in iBM1 and BMS^+^ sand flies. Together, these data indicate that *sherp* is not a reliable candidate for the detection of infectious sand flies and emphasize the relevance of conducting studies in vector sand flies to validate gene expression of candidates identified under culture conditions.

Blood feeding is a prominent event that recurs throughout the life span of sand flies. If we consider that the sand fly takes a blood meal every 5–6 days^[8]^, and that blood takes up to 3–4 days to be digested, then the parasites are mostly in constant contact with blood. Despite this intimate association between the blood fed state and *Leishmania*, fundamental information about the physical whereabouts of the parasites within the midgut following the intake of a subsequent bloodmeal and de novo synthesis of a PM remains unclear. We hypothesize that upon ingestion of blood, the newly synthesized PM may potentially trap a subset of parasites inside the new bloodmeal while others remain associated with the midgut lumen creating two subpopulations that are experiencing very distinct environments. This is supported by our data, where iBM1pp and BMS^+^pp show differences in gene expression making the toolkit possible. Knowing that blood profoundly affects the development of *Leishmania* parasites^[9, 29]^, establishing whether these two subpopulations occur and whether they are differentially reprogrammed would reveal novel and important aspects of host-parasite-vector interactions. For example, understanding the distribution of midgut-residing parasites in the context of recurrent blood meals may have potential implications for how hybrids, reported from natural isolates^[30]^, may form.

Throughout this study we investigated the expression of our target genes, *HPB* and *sherp*, in midgut-residing parasites after feeding on an artificial membrane or a sick hamster. As reported previously^[9]^, our data established a lower iBM1 parasite count estimate for naturally compared to artificially fed sand flies, emphasizing the need to verify translational tools in a more natural system. The toolkit performed well, with a predictive accuracy above 80%, in both artificial and naturally fed groups demonstrating the feasibility of PEARL in a natural setting.

In our controlled laboratory experiments, exposing the sand flies to a membrane feed or a sick animal takes a minimum of 1–2h, hence our earliest timepoint is indicated as ≤2h. Practically, investigators are unlikely to encounter amastigotes in sand flies from field captures unless a rare specimen is aspirated as it feeds on an infected animal. Even in this situation, the time to sorting and processing may add hours to the time of collection. This is relevant from a translational perspective where we expect that the vast majority of field collected blood fed sand flies will contain parasites that have been developing in the midgut for 8h or longer.

There are several limitations to our study. Our initial objective was to identify genes that were specifically upregulated in iBM1pp and BM3^+^pp to distinguish a specimen that fed on a reservoir animal from an infectious specimen, respectively. Unfortunately, though RNAseq of pooled sand flies revealed a few upregulated genes in iBM1 parasites, all failed validation in RT-qPCR of individual sand flies emphasizing the inadequacy of candidate selection based on RNAseq alone. Importantly, on a transcriptional level, BM2pp and BM3pp showed near identical expression of *HPB* and *sherp*. Future work is needed to mine better targets, not necessarily based on gene expression, that may be able to perform better at distinguishing iBM1pp, BM2pp, and BM3^+^pp thus reliably identifying infectious specimens in addition to reservoir animals. Further, the PEARL toolkit depends on finding enough *Leishmania*-infected blood fed specimens. This requires knowledge and expertise in where and when to trap sand flies in a study focus, and comprehensive collections to gather enough blood fed infected specimens from ecotypes of interest.

This work puts forward an innovative idea of how we can use sand flies to find unknown leishmaniasis reservoirs in endemic or emerging disease foci. The PEARL toolkit is versatile and can be deployed in any active leishmaniasis focus, including those in remote areas due to its field-friendly translational approach of preserving both DNA and RNA on filter paper. Considering that thousands of sand flies are captured and analyzed in numerous entomological studies of leishmaniasis foci, applying the PEARL toolkit to collected specimens has the potential to be the most efficient way of finding unknown animal reservoirs.

## Supplementary Material

Supplement 1

## Figures and Tables

**Table 1.** *sherp* locus architecture and copy number

Species (assembly)	*sherp* copies	Locus / arrangement	Gene IDs
*L. donovani* (BPK282A1)	1	Chr 23 (Ld23_v01s1): single copy at 506,195–506,365 (-)	LdBPK_*sherp* (manually added; unannotated in TriTrypDB-68)
*L. infantum* (JPCM5)	2 (tandem)	Chr 23 (LinJ.23): identical copies ~4 kb apart, 510,745–510,918 and 514,920–515,093 (-)	LINF_230018650, LINF_230018800 (100% identical)

**Table 2.** Top ten transcripts differentially expressed at the initial infected blood meal (iBM1) versus BM3.

Rank	Gene ID	Log_2_ fold change	adjusted *p* value
1	LdBPK_310370.1	2.5	5.6E-17
2	LdBPK_262710.1 (*HPB*)	2.5	1.1E-13
3	LdBPK_262600.1	2.5	1.6E-14
4	LdBPK_040160.1	1.9	2.1E-11
5	LdBPK_061320.1	1.5	4.7E-19
6	LdBPK_170690.1	3.2	2.7E-11
7	LdBPK_081190.1	−1.1	8.3E-11
8	LdBPK_323820.1	1.8	8.6E-11
9	LdBPK_050900.1	2.4	1.7E-10
10	LdBPK_190840.1	2.6	2.9E-10

iBM1, first infected blood meal; BM3, day 10.

**Table 3.** Percent identity of *HPB*, *sherp,* and *ssu rRNA* genes across *Leishmania* species. Alignment performed against available *Leishmania spp.* genomes.

Gene	Description	GenBank Accession	Nucleotide Identity
***HPB***	***Leishmania donovani hypothetical protein, unknown function (LDBPK_262710.1), partial mRNA***	**XM_003861807**	**100%**
	*Leishmania infantum JPCM5 hypothetical protein, unknown function partial mRNA*	XM_001470568	99.8%
	*Leishmania major strain Friedlin hypothetical protein partial mRNA*	XM_001684223	94.6%
	*Leishmania mexicana MHOM/GT/2001/U1103 hypothetical protein, unknown function partial mRNA*	XM_003876522	91.8%
***Sherp***	*Leishmania donovani small hydrophilic endoplasmic reticulum-associated protein*	Not annotated	-
	***Leishmania infantum JPCM5 small hydrophilic endoplasmic reticulum-associated protein***	**XM_003392466.1**	**100%**
	*Leishmania major strain Friedlin small hydrophilic endoplasmic reticulum-associated protein (sherp) (SHERP), partial mRNA.*	XM_001683388.1	90.2%
	*Leishmania mexicana MHOM/GT/2001/U1103 small hydrophilic endoplasmic reticulum-associated protein (sherp) partial mRNA*	XM_003875689.1	92.5%
***ssu rRNA***	*Leishmania donovani 18S ribosomal RNA gene, complete sequence (LdBPK_27rRNA6.1)*	GQ332356	99.7%
	*Leishmania infantum JPCM5 18S ribosomal RNA (SSU) RNA rRNA*	LINF_270031000-T1	99.7%
	***Leishmania major Friedlin 18S ribosomal RNA (SSU) RNA***	**XR_002460813**	**100%**
	*Leishmania mexicana 18S ribosomal RNA gene, complete sequence*	GQ332360	99.9%

Bolded, reference gene.

**Table 4.** Test-set classification performance of the linear discriminant analysis (LDA) classifier of iBM1 from BMS^+^ in membrane-fed and hamster-fed sand flies. For the F-measure, precision and recall metrics, the BMS^+^ samples are treated as “positive” events.

Infection	Predictors	Accuracy	F-measure	Precision	Recall
Artificial	*HPB+sherp*	0.87	0.89	0.92	0.85
Artificial	*HPB*	0.81	0.84	0.87	0.80
Artificial	*sherp*	0.87	0.89	0.92	0.85
Hamster	*HPB+sherp*	0.82	0.86	0.94	0.79
Hamster	*HPB*	0.79	0.82	0.93	0.74
Hamster	*sherp*	0.79	0.82	0.93	0.74

## Data Availability

All normalized and raw RNA seq data comparing BM1 vs BMS^+^ will be deposited at GitHub depository and available upon acceptance. All scripts used for data processing and analysis will be available at GitHub depository upon acceptance.
