# The PERL toolkit: Using sand flies to identify reservoirs of leishmaniasis

**DOI:** 10.64898/2026.06.27.734966

**Published:** 2026-09-09

**Authors:** Eva Iniguez, Patrick Huffcutt, Tiago Donatelli Serafim, Pedro Cecilio, Serena Doh, Aaron Pugh, Johannes Doehl, Claudio Meneses, Ben Lambert, Jesus G. Valenzuela, Shaden Kamhawi

## Abstract

Animal reservoirs remain unknown in emerging and most endemic leishmaniasis foci hindering control efforts. Here, we developed a field-applicable PERL (Phlebotomines Establish Reservoirs of *Leishmania* ) toolkit using individual blood fed sand flies (IBF) to identify leishmaniasis reservoirs. Using IBF, we optimized DNA and RNA co-extraction and parasite detection of ≥1 parasite/s by kDNA qPCR and *ssu rRNA* RT-qPCR. We then screened IBF for expression of two parasite genes, *sherp* and *HPB* , identified by RNAseq as having low-to-absent expression in IBF given a first *Leishmania donovani* -infected blood meal (IBF-iBM1) and high expression in specimens provided subsequent uninfected blood meals (IBF-BMS ^+^ ). Linear discriminant analysis of target gene expression classified iBM1 parasites with a predictive accuracy of ∼87% and ∼82% in membrane- or naturally-fed on hamsters sand flies, respectively. By determining the blood source in specimens determined as IBF-iBM1, the PERL toolkit provides an innovative and practical approach to identification of leishmaniasis reservoirs.

## Full Text Availability

The license terms selected by the author(s) for this preprint version do not permit archiving in PMC. The full text is available from the preprint server.

