# Supplementary material for: The PEARL toolkit: Using sand flies to identify leishmaniasis animal reservoirs
Source: bioRxiv. 2026 Jun 29:2026.06.27.734966. Preprint. [Version 1] doi: 10.64898/2026.06.27.734966 (PMC13345310; doi:10.64898/2026.06.27.734966)
Supplement: Supplement 1 [file media-1.pdf]

Supplemental material

The PEARL toolkit: Using sand flies to identify leishmaniasis animal reservoirs

Eva Iniguez[1#], Patrick Huffcutt[1#], Tiago Donatelli Serafim[1], Pedro Cecilio[2], Serena Doh[1], Aaron Pugh[1], Johannes Doehl[1], Claudio Meneses[1], Ben Lambert[3,4], Jesus G. Valenzuela[1], and Shaden Kamhawi[1*]

[1] Vector Molecular Biology Section, Laboratory of Malaria and Vector Research, National Institute of Allergy and Infectious Diseases, National Institutes of Health; Rockville, MD, USA
[2] Vector Biology Section, Laboratory of Malaria and Vector Research, National Institute of Allergy and Infectious Diseases, National Institutes of Health, Rockville, MD, USA
[3] Department of Statistics, University of Oxford, Oxford OX1 3LB, United Kingdom
[4] Pandemic Sciences Institute, University of Oxford, Oxford OX1 3LB, United Kingdom

#Equal contribution
*Correspondence: skamhawi@niaid.nih.gov

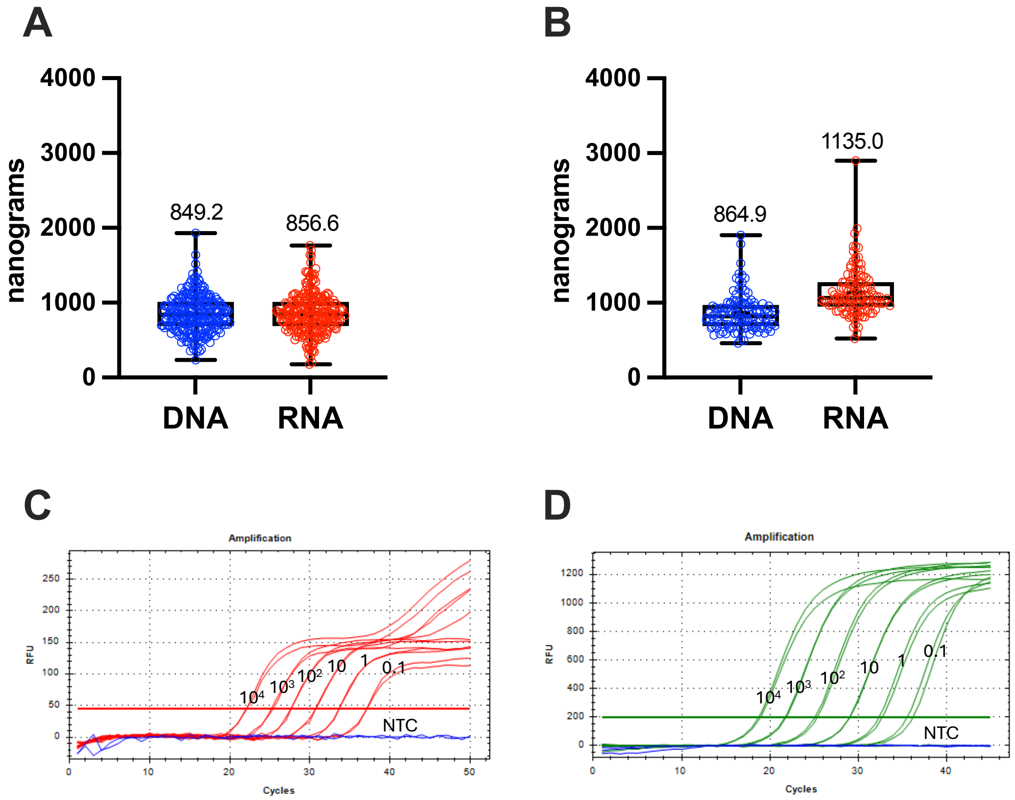

Figure S1. Co-extraction of DNA and RNA from individual blood fed sand flies and generation of standard curves. (A,B) Quantity of DNA and RNA extracted from *Leishmania*-infected midguts collected at ≤2 to 48 hours post-infection and preserved on Whatman 903 Protein Saver cards. Sand flies were either fed on a membrane (A) or a clinically ill hamster (B). Nucleic acids were extracted from samples stored at room temperature for up to 9 months. Bar, mean ± 95% CI. Each data point, an individual blood fed sand fly midgut quantified by nanodrop. (C,D) Representative amplification plots of a standard curve targeting kDNA by qPCR (C) or *ssu rRNA* by RT-qPCR (D). A relative fluorescent unit threshold was set for all the plates at 45 for (C) and 200 for (D). NTC, non-template control (blue).

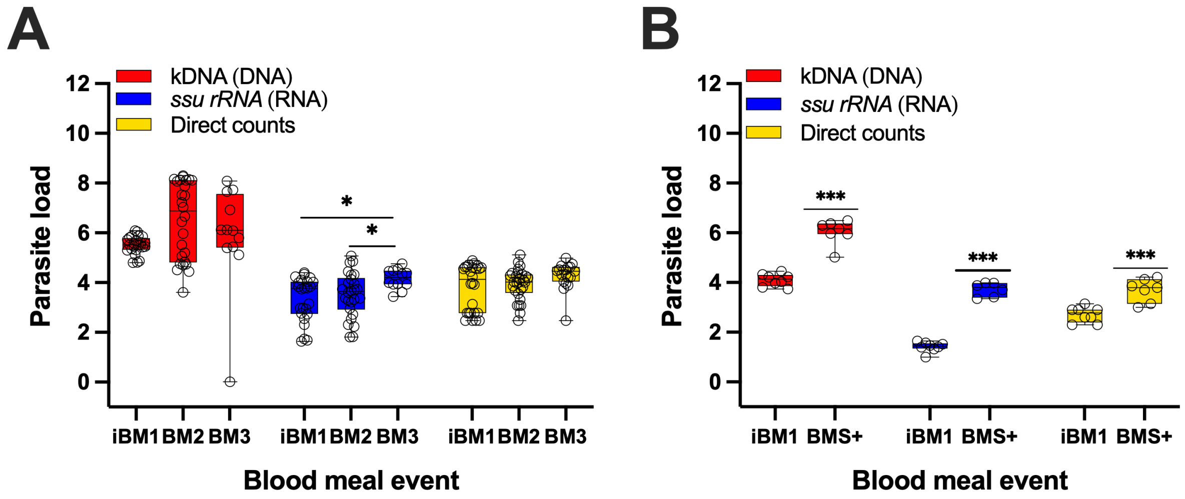

Figure S2. Validation of molecular targets for parasite quantification by comparison with microscopy counts in individual fed sand flies. (A,B) Parasites were molecularly quantified using a probe-based qPCR targeting kinetoplast DNA (kDNA), or RT-qPCR targeting the constitutively expressed parasite gene, *ssu rRNA*, and compared to direct counts by microscopy for sand flies fed on a membrane (A) or a clinically ill hamster (B). A standard curve was included in each plate to calculate the parasite load per sample. Cumulative data of two independent experiments (A), or one experiment (B). Bar, mean ±95% CI. Mann-Whitney (A) or Kruskal Wallis (B) test. A p value of ≤0.05 was considered significant, ***$p < 0.001$, *$p < 0.05$. For kDNA and *ssu rRNA*, each data point represents the mean of an IBF midgut ran in duplicate. iBM1, first infected blood meal; BM2, second uninfected blood meal on day 5-6; BM3, third uninfected blood meal on day 10-12; BMS^{+}, subsequent uninfected blood meal on day 10.

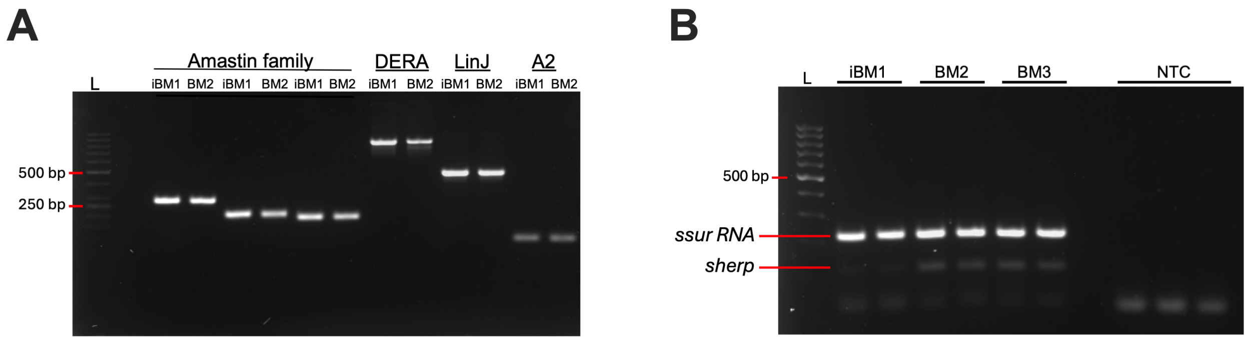

Figure S3: Previously characterized stage-specific genes are expressed across other parasite stages in the sand fly midgut. (A,B) Screening of amastigote genes (A), and *sherp* (B) in individual membrane fed sand flies by RT-PCR. Housekeeping gene *ssu rRNA* was screened in parallel (B). Representative samples from two independent experiments are shown; NTC, non-template control; L, ladder; iBM1, infected blood meal on day 1; BM2, second uninfected blood meal on day 6; BM3, third uninfected blood meal on day 12.

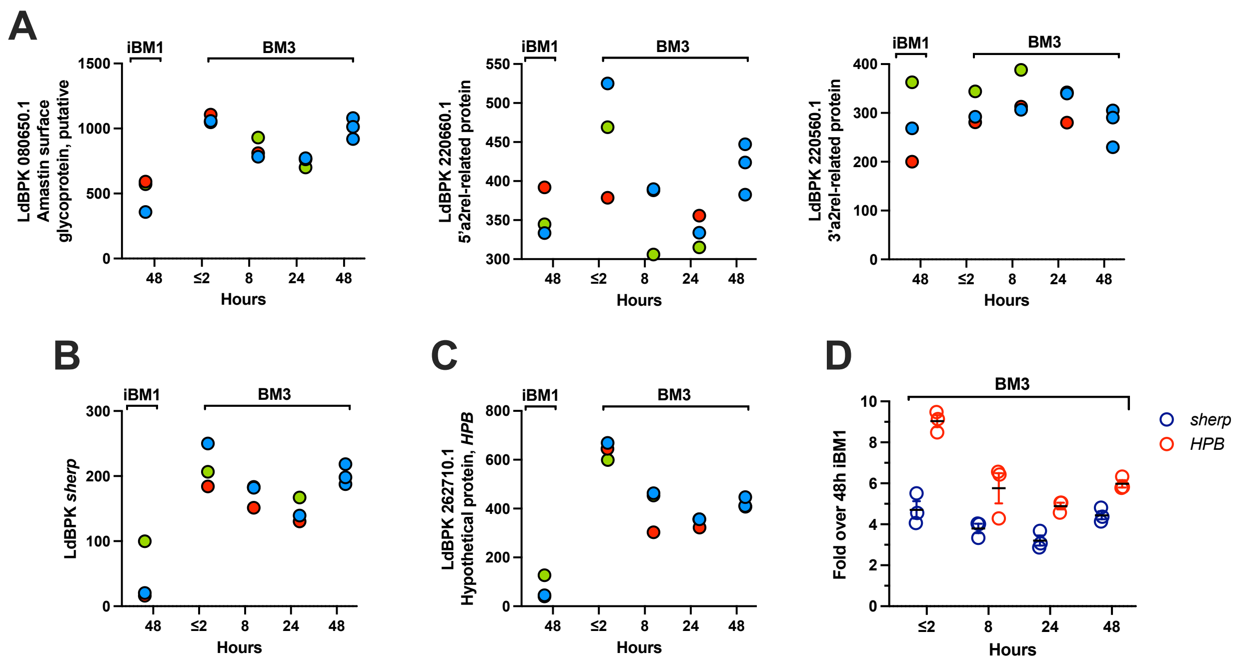

Figure S4. Discovery of *HPB,* a novel gene that distinguishes parasites residing in the sand fly midgut during the first infected blood meal from those in subsequent blood meals. (A-C) Bulk RNA-seq expression of the amastin gene and two members of the A2 gene family (A), *sherp* (B), and *HPB,* a novel target gene coding for a hypothetical protein (LdBPK_262710.1) (C). (D) Comparison of expression levels between *HPB* and *sherp*. Pools of 20 (iBM1), or 5 (BM3) fed midguts were collected per time point at 48 hours after iBM1, and ≤2, 8, 16, and 48 hours after BM3. Data from 3 independent experiments are shown. Graphs show the CPM (counts per million) representing the average gene expression level per experiment (A-C) or fold-change over 48h iBM1 (D). iBM1, first infected blood meal; BM3, a subsequent uninfected blood meal given on day 10 post infection.

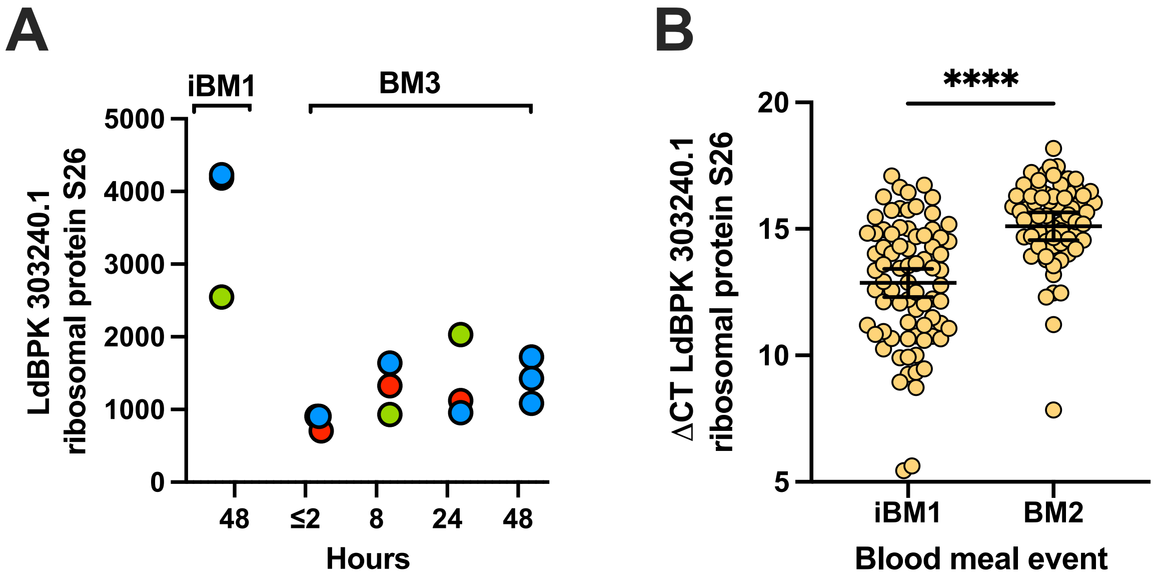

Figure S5. Validation of the most upregulated gene in the first infected blood meal compared to subsequent blood meals. (A) Bulk RNA-seq expression of gene LdBPK_303240.1, a ribosomal protein S26 upregulated in the iBM1pp. Pools of 20 (iBM1), or 5 (BMS^{+}) fed midguts were collected per time point at 48 hours after iBM1, and ≤2, 8, 16 and 48 hours after BM3. Data from 3 independent experiments are shown. Graphs show the CPM (counts per million) representing the average gene expression level per experiment. (B). Gene validation by qRT-PCR. ∆CT for each gene was calculated by subtracting the mean CT value of a midgut fed on uninfected blood from the mean CT value of the infected sample; Bar, mean ± 95% CI are shown; Mann-Whitney test. Cumulative data of two independent experiments are shown. Each data point represents the mean of an IBF midgut ran in duplicate. iBM1, first infected blood meal; BM2, a second uninfected blood meal on day 6; BM3, a subsequent uninfected blood meal on day 10.

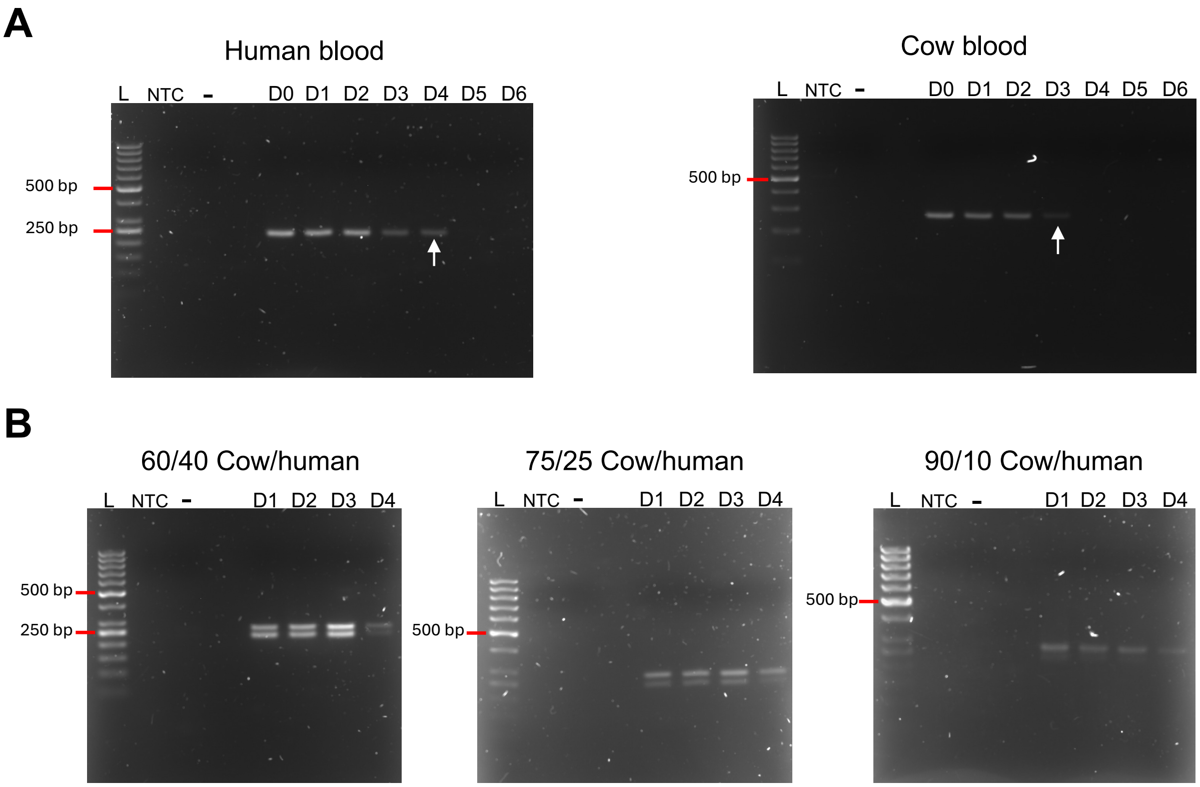

Figure S6. Detection of the blood meal source from sand flies fed on single or multiple hosts. (A,B) PCR amplification of DNA from individual sand flies fed on a single host (human or cow) (A) or fed on a human/cow blood meal mixed at different ratios (B). Samples were collected right after feeding and daily thereafter up to day 6 post-feeding. Gel shows the distinct band sizes corresponding to cytochrome b (*cytb*) and cytochrome c oxidase subunit I (COI) of vertebrate hosts (cow, 263bp, *cyb*; human 228bp, COI). Negative controls: NTC, non-template control; -, DNA from an experimental sand fly fed on rabbit blood; L, ladder. Arrowhead, last day of detectable blood.
